# XBP1 impacts lung adenocarcinoma progression by promoting plasma cell adaptation to the tumor microenvironment

**DOI:** 10.3389/fgene.2022.969536

**Published:** 2022-08-24

**Authors:** Zhaoqian Zhong, Junhao Wang, Qizheng Han, Hong Lin, Haihua Luo, Danyan Guo, Yong Jiang, Aihua Liu

**Affiliations:** ^1^ Guangdong Provincial Key Laboratory of Proteomics, State Key Laboratory of Organ Failure Research, Department of Pathophysiology, School of Basic Medical Sciences, Southern Medical University, Guangzhou, China; ^2^ Department of Respiratory and Critical Care Medicine, Nanfang Hospital, Southern Medical University, Guangzhou, China

**Keywords:** x-box binding protein 1, lung adenocarcinoma, tumor microenvironment, plasma cells, alternative splicing, endoplasmic reticulum stress

## Abstract

**Background:** The activation of X-box binding protein 1 (XBP1) plays an essential role in the unfolded protein response (UPR) of the endoplasmic reticulum (ER). XBP1 is commonly expressed in various tumors and is closely related to tumorigenesis and progression. However, the role of XBP1 in lung adenocarcinoma (LUAD), especially the prognostic value of its alternative splicing isoforms, remains largely unknown.

**Methods:** The LUAD datasets were retrieved from the The Cancer Genome Atlas, ArrayExpress and Gene Expression Omnibus. GEPIA2 and meta-analysis were employed to explore the prognostic value, and bioinformatics analysis with the TIMER2.0 database was used to investigate immune cell infiltration. We performed single-cell analyses to identify cell types with high XBP1 expression. In addition, polymerase chain reaction (PCR) and DNA sequencing were performed to verify the authenticity of the new spliceosome.

**Results:** In this study, we found that high expression of XBP1 was significantly associated with a good prognosis, and XBP1 expression was significantly positively correlated with B cell infiltration in LUAD. In addition, we found that high-level expression of a novel splicing isoform, XBP1 (XBP1-003), improved the prognosis of LUAD. Protein structural analysis demonstrated that XBP1-003 has several specific protein domains that are different from those of other XBP1 isoforms, indicating a unique function of this isoform in LUAD.

**Conclusion:** All these results suggest that XBP1 plays an antitumorigenic role in LUAD through alternative splicing, which may be related to the adaptation of plasma cells. This sheds new light on the potential strategy for LUAD prognosis evaluation and immunotherapy.

## Introduction

According to global cancer statistics, lung cancer has become the most prevalent cancer in the world and the leading cause of cancer-related death (Siegel et al., 2020). Lung cancer is mainly divided into small cell lung cancer (SCLC) and non-small-cell lung cancer (NSCLC). NSCLC accounts for 85% of all lung cancer cases and is frequently diagnosed at advanced stages (stage III or IV) due to the lack of obvious symptoms for patients in the early stages (stage I or II) (Osmani et al., 2018). Unfortunately, conventional therapeutic strategies for postoperative chemotherapy have limited effects on patients with advanced NSCLC (Gettinger and Lynch, 2011). According to histopathology, NSCLC is typically divided into two major subtypes: lung adenocarcinoma (LUAD) and lung squamous cell carcinoma (LUSC) (Faruki et al., 2017). LUAD is the most common subtype and accounts for more than 50% of all NSCLC cases (Travis, 2011).

It has been reported that the endoplasmic reticulum stress (ERS) was induced by lipin-1 gene silencing in LUAD cells, indicating that ERS is involved in the development of LUAD (Fan et al., 2018). The unfolded protein response (UPR) is defined as ERS caused by the accumulation of misfolded proteins in the endoplasmic reticulum (ER) lumen to maintain ER homeostasis, which plays a central role in the development and function of secretory cells (Moore and Hollien, 2012). Adaptation to ERS activates the UPR mechanism with the ER transmembrane proteins inositol requiring enzyme 1 alpha (IRE1α), activating transcription factor 6 (ATF6) and protein kinase R-like endoplasmic reticulum kinase (PERK). Downstream signaling then enhances proper protein folding, reduces the rate of protein biosynthesis, and promotes cell survival in an attempt to restore homeostasis (Hu et al., 2015).

As an alternative splicing transcription factor in the regulation of the UPR during ERS, X-box binding protein 1 (XBP1) is commonly expressed in various tumors and closely related to tumorigenesis and tumor progression (Chen et al., 2020). Notably, among the three established UPR pathways involved in persistent ERS resulting from the complex tumor microenvironment (TME), IRE1α-XBP1 signaling is the most conserved pathway and has been implicated in tumorigenesis and progression (Shi et al., 2019). Accumulating evidence suggests that the IRE1α-XBP1 pathway plays a critical role in the development of various cancers, and in particular, the levels of XBP1 isoforms may be critical in the fate determination of cancer cells (Davies et al., 2008), but the specific mechanism remains to be investigated.

In the clinic, immunotherapy has been used as an alternative to chemotherapy and radiotherapy. For example, inhibition of the immune checkpoint proteins PD-1 and PD-L1 reduces the effect of host antitumor immunity mediated by the TME (Iglesia et al., 2016). In addition, a previous study reported that immune cell infiltration in the TME was related to the survival of patients with solid tumors (Wang et al., 2020). Several studies on immune cell infiltration demonstrated that high levels of B cells in tumors perfectly predict overall survival in several types of tumors, including LUAD (Iglesia et al., 2016). As an independent risk factor, infiltrated B cells were used to predict the outcome of LUAD (Zheng et al., 2020). However, the reason for different infiltration of tumor-infiltrating B cells (TIBs) in patients remains terra incognita. Several reports have demonstrated a prominent and distinct association between antibody isoforms of TIBs and the survival of LUAD patients carrying specific driver mutations (Isaeva et al., 2019). Notably, in addition to the production of antibodies, TIBs act as antigen-presenting cells (APCs) to regulate cellular innate immunity in the TME and promote antigen-specific immune responses by suppressing tumor progression (Ritchie et al., 2004).

Tumor-infiltrating B cells and plasma cells (PCs) are believed to play an important role in the TME. On the one hand, TIBs produce antibodies, stimulatory cytokines and chemokines to enhance the T cell response; on the other hand, TIBs serve as APCs and cooperate with T cells and dendritic cells to form tertiary lymphoid structures (TLSs), thereby sustaining long-term immunity (Nelson, 2010). Furthermore, the high density of tumor-associated B cells in TLSs is a biomarker for favorable overall survival in NSCLC (Germain et al., 2014). Therefore, it is worth studying the function of TIBs in tumors.

Because of the drastic ERS response caused by the accelerated synthesis and secretion of immunoglobulins (Ig) from PCs, protein homeostasis is critical for PC survival, but the molecular mechanisms are still unknown. Interestingly, of the components of the three UPR pathways, only IRE1α has the capability to enhance PC function by alleviating ERS and promoting protein folding, thus mediating the terminal differentiation and maturation of PCs (Jiang et al., 2015). Previous studies showed that XBP1 acts as a central transcription factor of the IRE1α signaling pathway to maintain ER homeostasis in response to the increased protein production accompanying PC maturation (Ali et al., 2011). Coincidentally, XBP1 has been demonstrated to be downstream of Blimp-1, which upregulates the expression of most PC-specific genes to increase protein synthesis in PCs during PC differentiation (Shaffer et al., 2004). In addition, XBP1 increases cell size, mitochondrial mass and function, ribosome number, and lysosome content, indicating that XBP1 coordinates various changes in cell structure and function. However, the detailed mechanism involved in this complicated process is worth illuminating.

In this study, we attempted to clarify the function of XBP1 and the prognostic value of high expression of XBP1 alternative splicing isoforms in LUAD patients. We found that XBP1 plays an antitumorigenic role in LUAD through alternative splicing, which may be associated with B cell infiltration.

## Materials and methods

### Cell culture

The MM cell line MM.1S (human, female, RRID: CTCC-004-0048) was maintained in RPMI Medium 1640, 10% fetal bovine serum (FBS), TransMycoPre (1:1000, RRID: FM501, TRANS) at 37°C in 5% CO_2_.

### Single-cell analysis of lung tissue from lung adenocarcinoma patients

We used Seurat v4.0 (Hao et al., 2021) R package to analyze the single-cell RNA-seq raw matrix, which was downloaded from GEO (GSE131907) and included 22 cases of LUAD and distant normal lung tissue (Kim et al., 2020). After reintegration of the LUAD (tLung) and Normal (nLung) samples, we extracted B lymphocytes and used multimodal reference mapping to identify the subpopulations of B lymphocytes. The pathway score was calculated using the AddModuleFeature function and projected it onto the single-cell data. The gene sets (GOBP: negative regulation of response to endoplasmic reticulum stress and GOBP: protein folding) were downloaded from the Molecular Signatures Database (MSigDB v7.5.1) (Subramanian et al., 2005).

### Expression analysis of X-box binding protein 1

The expression of XBP1 in different tumor tissues (T) and adjacent peritumor tissues setting as normal (N) was identified in the Gene Expression Profiling Interactive Analysis 2 (GEPIA2; http://gepia2.cancer-pku.cn/), which integrates RNA-seq data from The Cancer Genome Atlas (TCGA) and Genotype-Tissue Expression (GTEx) databases (Tang et al., 2019). GEPIA2 was also used to identify the expression and usage of XBP1 isoforms in LUAD. The Human Protein Atlas (HPA; www.proteinatlas.org) database was used to display the XBP1 mRNA distribution expressed as the mean protein-coding transcripts per million (pTPM) in each individual sample from the GTEx dataset (Uhlen et al., 2017). The TIMER2.0 (http://timer.cistrome.org/) database is a comprehensive resource for the systematic analysis of immune infiltration in various tumor types (Li et al., 2020). We used the TIMER2.0 “gene-outcome” module to determine the clinical relevance of XBP1 gene expression in various tumors by using a Cox proportional risk model. A heatmap was used to show the normalized coefficients of genes in this model. Finally, we performed a meta-analysis of XBP1 expression using the online tool Lung Cancer Explorer (http://lce.biohpc.swmed.edu/lungcancer/metagenename.php), which contains 56 lung cancer datasets from the Sequence Read Archive (SRA) and Gene Expression Omnibus (GEO) databases, to verify the expression of XBP1 in LUAD (Cai et al., 2019).

### Prognosis analysis

The quartile of XBP1 expression was set as the cutoff to determine the high (>75%) and low (<25%) expression samples, and the GEPIA2 database was used to assess the effect of XBP1 expression on the overall survival (OS) of different tumor types. Based on survival information from the GEPIA2 database, we further examined the OS curves of the two different XBP1 transcripts XBP1-001 (ENST00000216037.10) and XBP1-003 (ENST00000405219.7) with the same cutoff settings. Log-rank *p* values and hazard ratios (HRs) with 95% confidence intervals (CIs) were calculated. Additionally, we performed a meta-analysis to verify the prognostic implications of XBP1 in LUAD using the Lung Cancer Explorer database, and the results were revalidated at the protein level with The Cancer Proteome Atlas (TCPA; http://tcpaportal.org/tcpa/index.html) database (Li et al., 2013). We used GEPIA 2021 (http://gepia2021.cancer-pku.cn/), a standalone extension with multiple deconvolution-based analysis for GEPIA (Li et al., 2021), to evaluate the influence of infiltrating immune cells, including B cells, CD4 T cells, CD8 T cells, endothelial cells, macrophages and natural killer (NK) cells, on the OS of LUAD patients.

### Gene enrichment analysis

Metascape (http://metascape.org) is a comprehensive portal combining functional enrichment, interactome analysis, gene annotation and member search to leverage more than 40 gene function annotation databases (Zhou et al., 2019). We used Metascape to perform enrichment analysis, including gene ontology (GO) and Kyoto Encyclopedia of Genes and Genomes (KEGG), on the top 100 genes with expression profiles similar to XBP1 obtained from GEPIA2.

### Protein-protein interaction network construction and module analysis

The top 100 genes with expression profiles similar to that of XBP1 were analyzed by Search Tool for the Retrieval of Interacting Genes/Proteins (STRING) (v11.0, https://string-db.org/) (Szklarczyk et al., 2021). Cytoscape was used to visualize the protein-protein interaction (PPI) networks, and molecular complex detection (MCODE) was used to screen core modules of the interacting networks, with the settings of degree cutoff = 2, node score cutoff = 0.2, k-core = 2, and max depth = 100. Metascape was used to perform analysis of the PPI networks with the mixture of the top 100 genes with profiles similar to that of XBP1, XBP1-001 and XBP1-003 in LUAD.

### Analysis of X-box binding protein 1 expression in infiltrated immune cells of lung adenocarcinoma

We used the GEPIA2021 database with the “subexpression” module to compare the expression of XBP1 in immune cells between LUAD and adjacent normal tissues from the CIBERSORT dataset and the EPIC dataset. Immune cells, including PCs, follicular helper T cells, resting NK cells, monocytes and neutrophils, in the CIBERSORT dataset as well as 5 cell types (B cells, CD4 T cells, macrophages, endothelial cells and cancer-associated fibroblasts) in the EPIC dataset were selected to visualize the expression of XBP1 with an interactive boxplot. The Immune Cell Gene Expression Atlas from the University of Tokyo (ImmuNexUT; https://www.immunexut.org/) is a gene expression and expression quantitative trait loci (eQTL) atlas of 28 types of immune cells isolated from 10 samples from patients with distinct human immune-mediated diseases, as well as samples from healthy donors (Ota et al., 2021). The expanding atlas consists of 9,852 immune cell samples from 416 donors. We employed the “gene” module of TIMER2.0 to visualize the correlation of XBP1 expression with the levels of multiple tumor-infiltrating lymphocytes (TILs), including B cells, PCs, endothelial cells, macrophages and monocytes. The Tumor Immune Single-Cell Hub (TISCH; http://tisch.comp-genomics.org) is a TME-related RNA-seq database that provides detailed cell type annotation at the single-cell level, enabling fast, flexible and comprehensive exploration of the TME (Sun et al., 2021). We further employed TISCH to analyze the heterogeneity of the TME in three datasets: NSCLC_EMTA6149, NSCLC_GSE127465 and NSCLC_GSE139555.

### Prediction of protein structure

Information on XBP1 splicing isoforms was collected from the Ensembl database (https://asia.ensembl.org/index.html) (Howe et al., 2021), and the protein sequences of XBP1-001 (ID: P17861) and XBP1-003 (ID: B1AHH1) were downloaded from the UniProt database (http://www.uniprot.org/) (The UniProt Consortium, 2021). The structural data files of XBP1-001 (59 aa-220 aa) and XBP1-003 (21 aa-135 aa) in PDB format were downloaded from ModBase (https://modbase.compbio.ucsf.edu/) (Pieper et al., 2014), which was followed by protein structure visualization with PyMOL software.

The protein domains of XBP1-001 and XBP1-003 were further predicted with PredictProtein (https://www.predictprotein.org/) based on their amino acid sequences (Bernhofer et al., 2021), and the top 20 clusters from the biological process (BP) category of GO terms were collected. Posttranslational modification (PTM) data, including phosphorylation and ubiquitination data, were acquired from the cBioPortal database (http://www.cbioportal.org) (Cerami et al., 2012). The RNA binding sites of XBP1 isoforms were predicted by the online tool catRAPID (http://s.tartaglialab.com/page/catrapid_group) (Armaos et al., 2021), and only the binding sites with an overall interaction score greater than 0.5 are displayed.

### Functional analysis and phenotypic determination of X-box binding protein 1

The relationship between gene function and XBP1 expression was analyzed by CancerSEA (http://biocc.hrbmu.edu.cn/CancerSEA/) (Yuan et al., 2019), which is a comprehensive database that aims to comprehensively decode distinct functional states of cancer cells at single-cell resolution. It provides a functional state atlas of cancer, involving 14 functional states, including stemness, invasion, metastasis, proliferation, epithelial–mesenchymal transition (EMT), angiogenesis, apoptosis, cell cycle, differentiation, DNA damage, DNA repair, hypoxia, inflammation and quiescence, of 41,900 cancer single cells from 25 cancer types. To further determine the association of Xbp1 expression and mouse phenotyping parameters, we selected the significant phenotypes (mortality/aging, homeostasis/metabolism or adipose tissue, skeleton, immune system or hematopoietic system, and vision/eye) from the International Mouse Phenotyping Consortium (IMPC; http://www.mousephenotype.org) (Dickinson et al., 2016) resource.

### Polymerase chain reaction and DNA sequencing

Total RNA from MM.1S cells was extracted using TRIzol reagent (#15596026, Invitrogen, Carlsbad, California, United States). Reverse transcription of total RNA was performed using HiScript III RT SuperMix for qPCR (Vazyme), and then the PCR products were amplified with KOD-Plus-Neo (RRID: KOD-401, TOYOBO) to verify the presence of XBP1-003. Amplification products were sent to Huada Genomics Institute (BGI, Guangzhou, China) for DNA sequencing. The primers were as follows: XBP1-003 forward primers 5′-ACT​CTC​TCG​TTA​GAG​ATG​ACC​AGA​GC-3′, and reverse primers 5′-CGA​ATT​AGT​TCA​TTA​ATG​GCT​TCC​AGC​T-3′.

## Results

### Gene expression profiles of X-box binding protein 1 in multiple tumor tissue types or healthy control tissues

**FIGURE 1:**
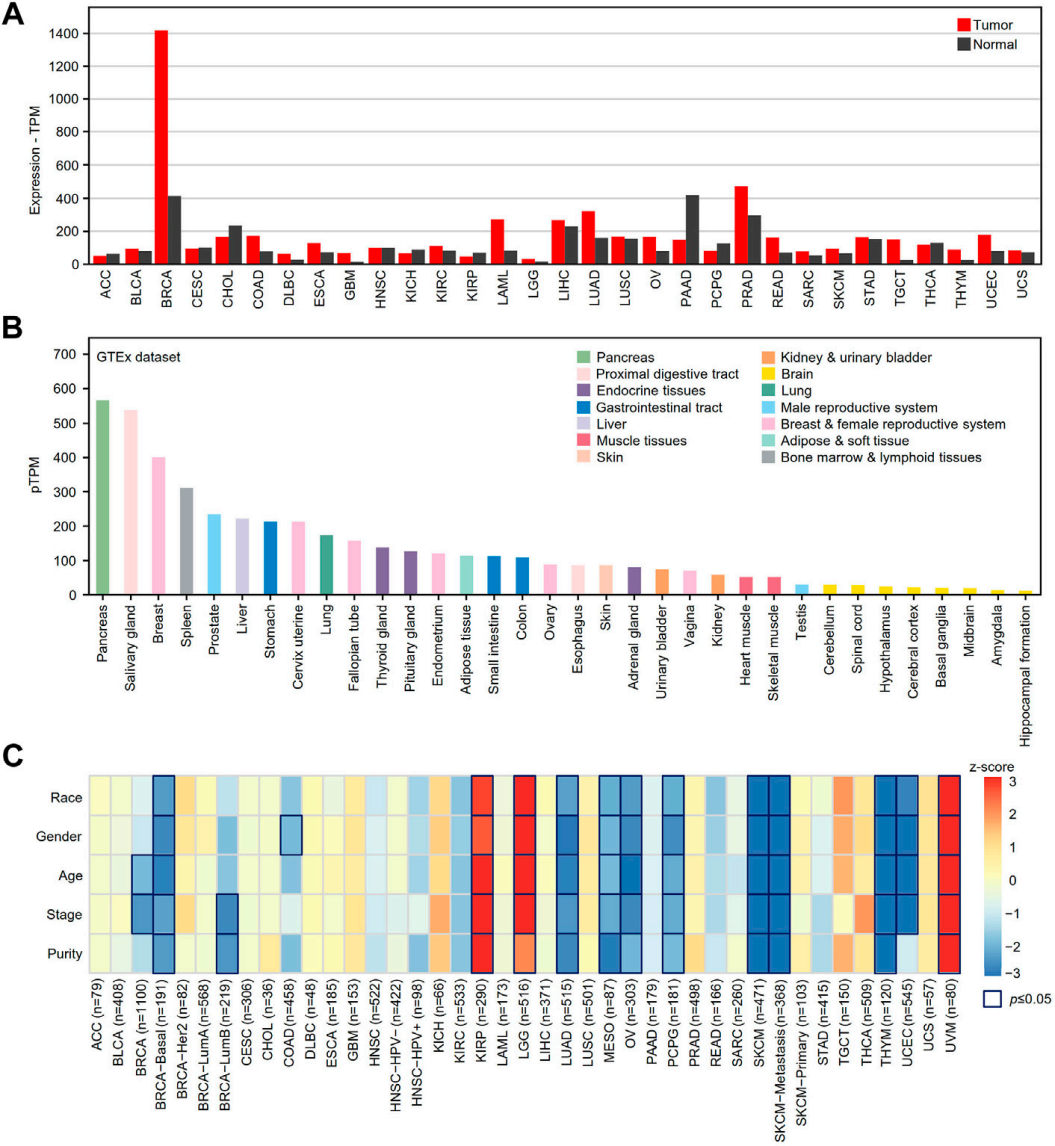
The mRNA expression analysis of XBP1 in various human cancer tissues. **(A)** XBP1 expression in different human cancer samples was determined by GEPIA2 in comparison with adjacent peritumor samples. **(B)** The expression profiles of XBP1 in healthy human tissues in HPA. Color-coding is based on tissue groups, each consisting of tissues with functional features in common. **(C)** The survival correlation of XBP1 expression adjusted by different clinical factors in the TIMER2.0 database. The result of *p* ≤ 0.05 is significant. Abbreviation: ACC, adrenocortical carcinoma; BLCA, bladder urothelial carcinoma; BRCA, breast invasive carcinoma; CESC, cervical squamous cell carcinoma (include endocervical adenocarcinoma); CHOL, cholangio carcinoma; COAD, colon adenocarcinoma; DLBC, lymphoid neoplasm diffuse large B-cell lymphoma; ESCA, esophageal carcinoma; GBM, glioblastoma multiforme; HNSC, head and neck squamous cell carcinoma; KICH, kidney chromophobe; KIRC, kidney renal clear cell carcinoma; KIRP, kidney renal papillary cell carcinoma; LAML, acute myeloid leukemia; LGG, brain lower grade glioma; LIHC, liver hepatocellular carcinoma; LUAD, lung adenocarcinoma; LUSC, lung squamous cell carcinoma; MESO, mesothelioma; OV, ovarian serous cystadenocarcinoma; PAAD, pancreatic adenocarcinoma; PCPG, pheochromocytoma and paraganglioma; PRAD, prostate adenocarcinoma; READ, rectum adenocarcinoma; SARC, sarcoma; SKCM, skin cutaneous melanoma; STAD, stomach adenocarcinoma; TGCT, testicular germ cell tumors; THCA, thyroid carcinoma; THYM, thymoma; UCEC, uterine corpus endometrial carcinoma; UCS, uterine carcinosarcoma; UVM, uveal melanoma.

The GEPIA2 online tool was used to determine the differential expression of XBP1 in tumor tissues and adjacent peritumor normal tissues. We found that XBP1 was highly expressed in the majority of tumors, including breast invasive carcinoma (BRCA), colon adenocarcinoma (COAD), esophageal carcinoma (ESCA), acute myeloid leukemia (LAML), ovarian serous cystadenocarcinoma (OV), prostate adenocarcinoma (PRAD), skin cutaneous melanoma (SKCM), testicular germ cell tumor (TGCT), thymoma (THYM), uterine corpus endometrial carcinoma (UCEC) and LUAD, but not pancreatic adenocarcinoma (PAAD) (Figure 1A).

Next, we examined the mRNA expression of XBP1 in various healthy tissues and organs to help us to understand the role of XBP1 expression in tumorigenesis and progression. As shown in Figure 1B, XBP1 was significantly highly expressed in glands such as the pancreas, salivary glands, and mammary glands, as well as organs with a gland-rich distribution such as the spleen, liver, stomach and lung, suggesting that XBP1 has a function closely related to protein secretion.

To further explore the impact of XBP1 on tumors, we evaluated the correlation between XBP1 expression and clinical factors of multiple tumors in the TIMER2.0 database. Interestingly, we found that XBP1 expression was significantly associated with the survival of some tumor patients after adjusting for clinical factors, including age, sex, race, stage and purity. After adjusting for tumor purity, the survival risk of XBP1 overexpression was significantly increased in kidney renal papillary cell carcinoma (KIPR), brain lower grade glioma (LGG) and uveal melanoma (UVM) but decreased in LUAD, mesothelioma (MESO), OV, pheochromocytoma and paraganglioma (PCPG), SKCM and THYM (Figure 1C). However, there was no significant association between XBP1 expression and survival risk in LUSC.

We further verified whether the expression of XBP1 is related to the prognosis of these tumors in the GEPIA2 database. We found that XBP1 overexpression was an unfavorable factor in terms of the survival of KIPR, LGG and UVM patients (Supplementary Figures S1A–S1C) but was associated with a good prognosis for UCEC, MESO, SKCM, and THYM patients (Supplementary Figures S1D–S1G). However, we found that XBP1 was only differentially expressed in LGG, UCEC, and THYM patients versus normal controls (Supplementary Figures S1H–S1J), indicating that XBP1 is an important regulator involved in the tumorigenesis and progression of these tumors.

### Association of elevated X-box binding protein 1 expression with a good prognosis in lung adenocarcinoma patients

**FIGURE 2:**
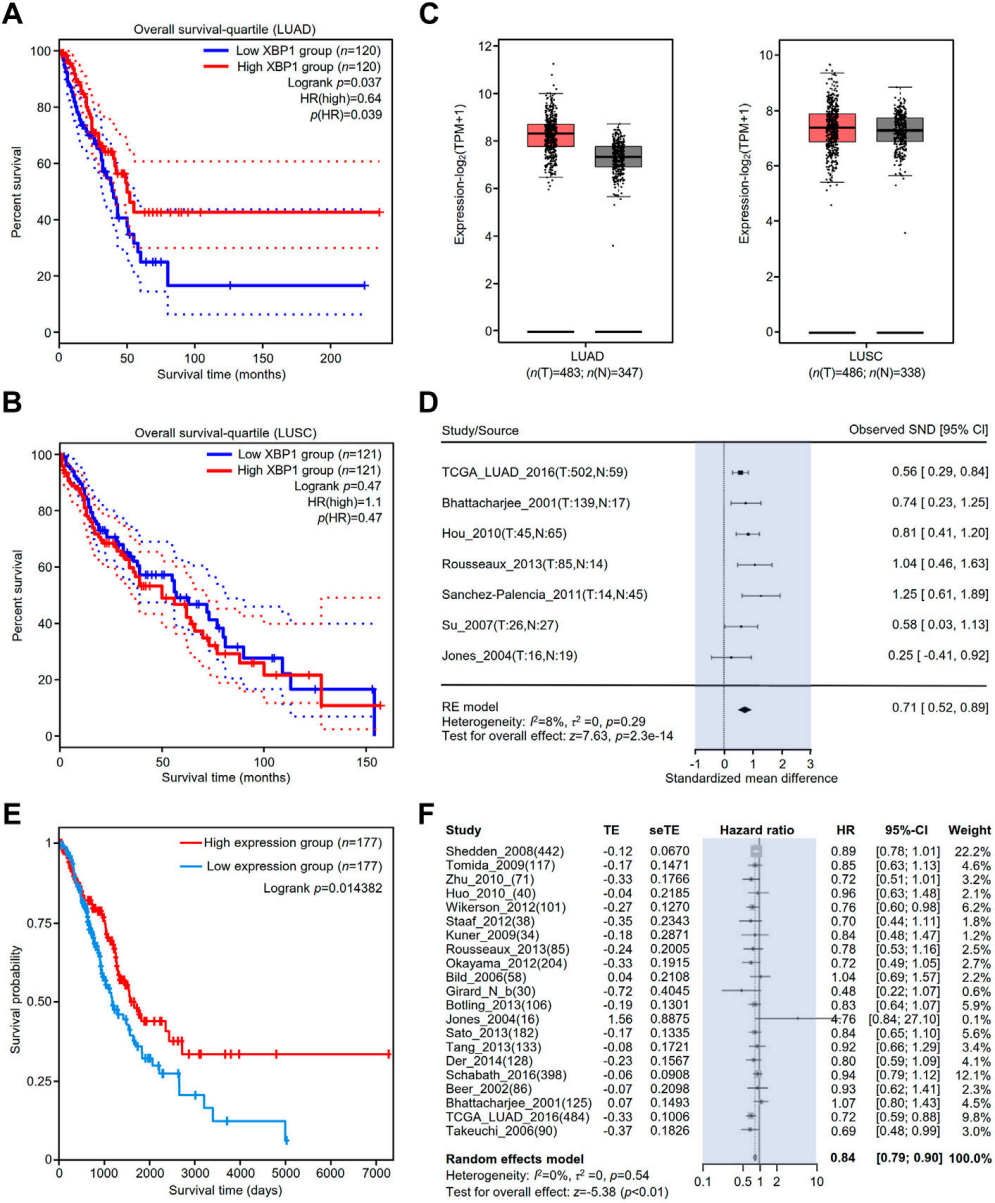
The prognostic value of XBP1 expressing in the lung tissues of patients with LUAD or LUSC. **(A,B)** The prognostic value of XBP1 mRNA level expressing in LUAD and LUSC patients was evaluated by GEPIA2. **(C)** Expressional boxplot of XBP1 in LUAD and LUSC based on GEPIA2. **(D,E)** The favorable prognosis of XBP1 overexpression in LUAD was verified by TCPA **(D)** and Lung Cancer Explorer database **(E)**. **(F)** Meta-analysis of XBP1 expression in LUAD from the Lung Cancer Explorer database.

To clarify the association between the expression of XBP1 and the prognosis of NSCLC, we evaluated the impact of XBP1 expression on the OS of LUAD and LUSC. We found that high XBP1 expression was significantly associated with good prognosis in LUAD (Figure 2A) but not OS in LUSC (Figure 2B). In line with this result, we also found that XBP1 mRNA expression was upregulated in LUAD but not LUSC (Figure 2C), indicating that XBP1 overexpression plays an important role in LUAD. Furthermore, we found that XBP1 expression was significantly higher in LUAD tissues than in adjacent normal lung tissues by meta-analysis (Figure 2D).

To validate the correlation between XBP1 overexpression and a favorable prognosis, we examined the prognostic value of XBP1 protein expression in LUAD using the TCPA database and found that LUAD patients with high levels of XBP1 protein had a favorable prognosis in comparison with those with low levels of XBP1 protein (Figure 2E), which is consistent with the result of XBP1 gene expression analysis in the GEPIA database. We further applied the online tool Lung Cancer Explorer to determine whether the high expression of XBP1 is beneficial to the survival of LUAD patients. The forest plot of the meta-analysis showed that high XBP1 expression was associated with good prognosis (Figure 2F). These findings indicate that a high level of XBP1 expression plays an antitumorigenic role in LUAD.

### The biological function of X-box binding protein 1 in lung adenocarcinoma and normal tissues

**FIGURE 3:**
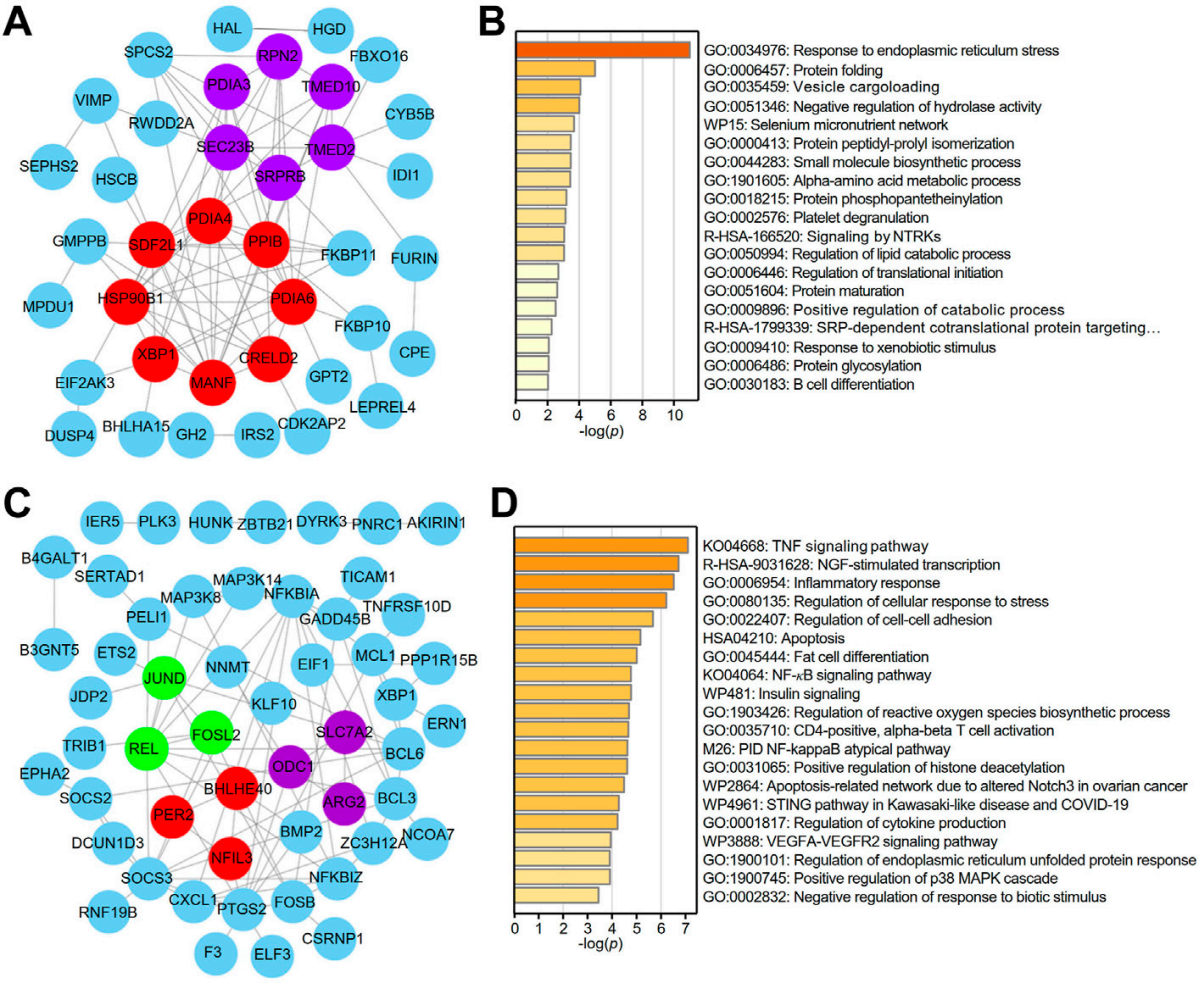
Identification of potential functions of XBP1 in LUAD. **(A)** The PPI network of top 100 genes with similar expression profile of XBP1 in LUAD. STRING and Cytoscape were used to analyze the PPI network and tow (red and purple) core modules were identified. **(B)** Metascape pathway enrichment analysis of top100 genes with similar expression profile of XBP1 in LUAD, colored by *p*-values. **(C)** The PPI network of top 100 genes with similar expression profile of XBP1 in adjacent peritumor normal tissues. Three (red, purple and green) core modules were identified by STRING and Cytoscape. **(D)** Top 20 clusters from Metascape pathway enrichment analysis of top 100 genes with similar expression profile of XBP1 in adjacent peritumor normal tissues, colored by *p*-values.

A PPI network of the top 100 genes with profiles similar to that of XBP1 in LUAD was generated using STRING, and two core (red and purple) modules were successfully identified with Cytoscape software (version: 3.8.2) (Figure 3A). To reveal the potential functions of XBP1 in LUAD, we performed enrichment analysis on the genes with profiles similar to that of XBP1 using Metascape and found that these genes were enriched in protein structure regulation-related GO terms, including protein folding, protein peptidyl-prolyl isomerization, protein phosphopantetheinylation, protein maturation, and protein glycosylation (Figure 3B). Additionally, we found that these genes were associated with B cell differentiation, suggesting that XBP1 might be involved in determining the localization of infiltrated B cells to regulate tumorigenesis and progression of LUAD (Figure 3B). Notably, these two core modules were mainly enriched in some important GO terms: the terms response to ERS and protein folding were enriched in the red module, and vesicle cargo loading was enriched in the purple module (Figure 3B), indicating that XBP1 plays an important role in misfolded protein processing induced by ERS in LUAD.

To clarify whether the PPI networks in normal tissues are different from those in LUAD tissues, we performed the same procedure for the analysis of the genes with profiles similar to that of XBP1 in normal tissues and found that both the PPI network and enriched GO terms related to these genes in normal tissues were significantly different from those in LUAD tissues. Interestingly, the three core modules (red, purple and green) were found to be associated with circadian rhythm, amino acid metabolic pathways and cytokine responses (Figure 3C). Importantly, GO enrichment analysis demonstrated that XBP1 was mainly involved in inflammation and stress-induced cell responses, including the inflammatory response, regulation of cellular response to stress, regulation of reactive oxygen species (ROS) biosynthetic process, and regulation of cytokine production. Consistently, KEGG analysis showed that some inflammation- or immune-related signaling pathways were highly enriched, such as the TNF signaling pathway, NF-*κ*B signaling pathway, STING pathway, and VEGFA-VEGFR2 signaling pathway (Figure 3D).

To further determine the correlation of XBP1 with the cellular process of LUAD, we performed single-cell analysis by CancerSEA and found that high XBP1 expression had a negative correlation with metastasis, invasion, angiogenesis and DNA damage in LUAD cells (Supplementary Figures S2A, S2B), which further suggests that XBP1 plays an antitumorigenic role in LUAD. In addition, we performed phenotyping tests with Xbp1 gene knockout mice to determine the link between Xbp1 and phenotypes and found that Xbp1 was involved in the regulation of clinical chemistry, body composition and eye morphology in mice (Supplementary Figure S2C), suggesting that Xbp1 plays an important role *in vivo*.

### Prognostic value of infiltrated PCs in lung adenocarcinoma with high X-box binding protein 1 expression

**FIGURE 4:**
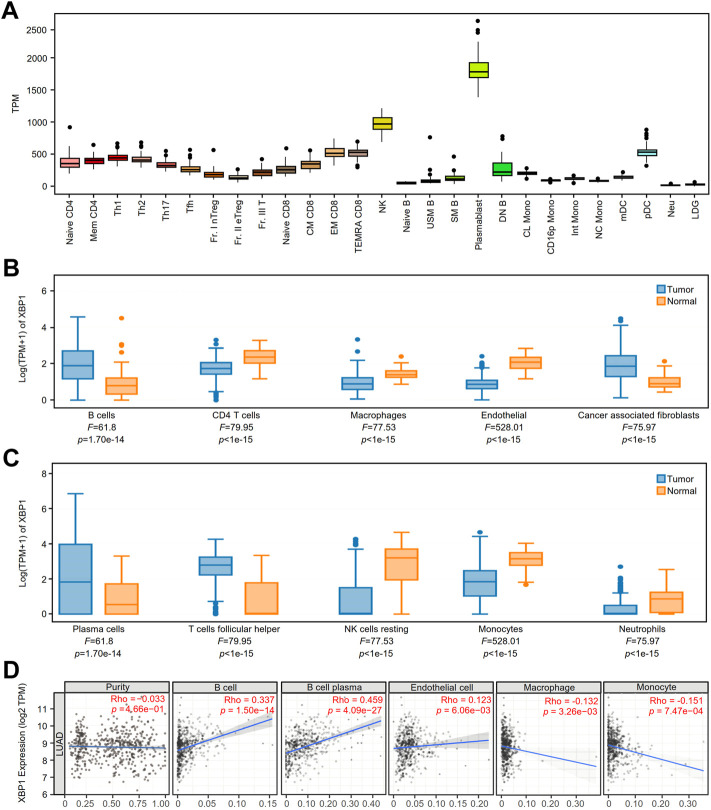
The expression of XBP1 in various immune cells and its association with immune infiltration in LUAD. **(A)** XBP1 expression atlas of immune cells of healthy individuals from the ImmuNexUT. **(B,C)** The expression of XBP1 in different immune cells in LUAD and adjacent peritumor normal tissues were analyzed based on EPIC **(B)** and CIBERSORT **(C)** using GEPIA 2021. **(D)** Correlative expression of XBP1 with LUAD purity and infiltration of immune cells, including B cells, plasma cells, endothelial cells, macrophages and monocytes in TIMER2.0.

We detected the expression of XBP1 in various immune cells under physiological conditions and found that XBP1 was mainly expressed in PCs (Figure 4A). Coincidentally, we found that XBP1 was selectively expressed in PCs in the NSCLC datasets EMTAB6149, GSE127465 and GSE139555 from the TISCH database (Supplementary Figures S3A–S3C).

To confirm the speculation that XBP1 affects the prognosis of LUAD through regulating the infiltration of PCs, we examined XBP1 expression and immune cell infiltration in LUAD using the GEPIA2021 database and found that XBP1 was mainly expressed on B cells and PCs in LUAD (Figures 4B,C). Subsequently, we investigated the correlation between XBP1 expression and TILs in LUAD and found that XBP1 expression had a significant correlation with the infiltration of B cells and PCs but not with tumor purity (Figure 4D), which suggests that XBP1 was mainly expressed in B cells and not in cancer cells. Interestingly, in the analysis of infiltrated immune cells (B cells, CD4 T cells, CD8 T cells, endothelial cells, macrophages and NK cells), only B cell infiltration had significant prognostic value in LUAD (Supplementary Figures S4A–S4F). Therefore, it is reasonable to assume that XBP1 exerts its antitumorigenic effects by affecting B cells in LUAD.

### Validation of high X-box binding protein 1 expression in infiltrating PCs

**FIGURE 5:**
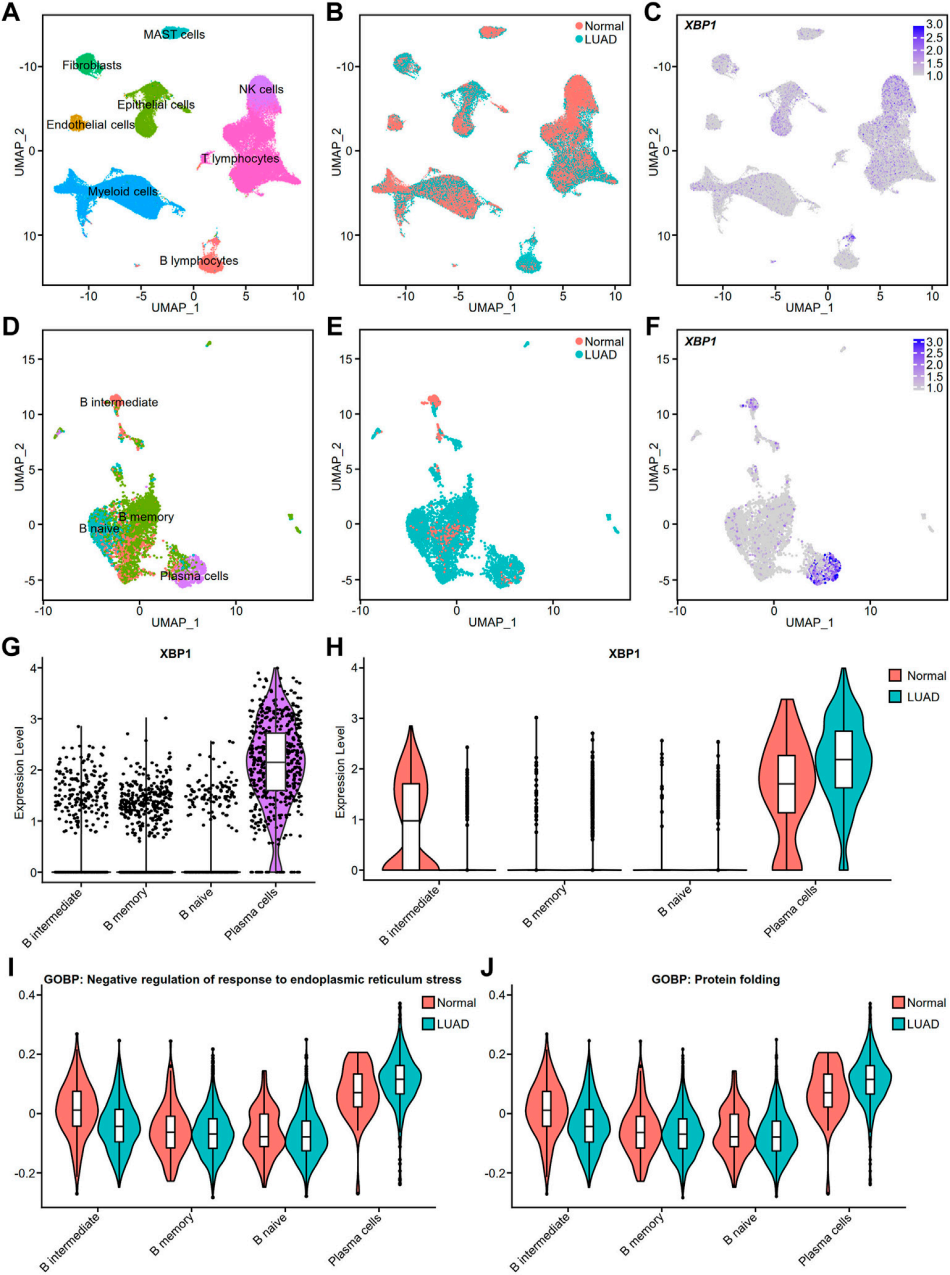
Cell type validation and XBP1 expression in LUAD. **(A)** Single-cell analysis of cell clusters from tumor tissue and distant normal tissue in 11 patients with LUAD. The human lung cell clusters were visualized by UMAP in different colors. **(B)** UMAP plot of lung cells with LUAD and Normal. **(C)** XBP1 expression on the UMAP plot of lung tissue. **(D)** Further single-cell analysis of detailed cell clusters from the B lymphocyte subset **(E)** LUAD and Normal B lymphocytes UMAP plot. **(F)** XBP1 expression on the UMAP plot of B lymphocytes. **(G,H)** Violin plot of XBP1 expression in various B lymphocyte subpopulations. **(I)** Violin plot of the gene sets involved in the negative regulation of endoplasmic reticulum stress expressed in various cell clusters of B lymphocytes. **(J)** Violin plot of the gene sets involved in the protein folding expressed in various cell clusters of B lymphocytes. Split-violin plots showing the expression of XBP1 in LUAD and Normal. A box plot is embedded in each violin plot.

To verify the cell types with high XBP1 expression, we performed a single-cell analysis of the single-cell RNA-seq raw matrix (GSE131907) downloaded from GEO. After computational processing, we captured 37,299 normal cells and 39,174 tumor cells that were divided into 8 distinct clusters, including MAST cells, Fibroblasts, Epithelial cells, NK cells, Endothelial cells, Myeloid cells, T lymphocytes and B lymphocytes (Figure 5A). Many cells from LUAD appeared to be increasing, with Fibroblasts, Epithelial cells and B lymphocytes being the clusters with the greatest increase in numbers (Figure 5B). We found that XBP1 was highly expressed in certain B lymphocytes (Figure 5C), so we extracted all 4,726 B lymphocytes and used multimodal reference mapping to identify the subpopulations of B lymphocytes (Figure 5D), confirming that a large number of B lymphocytes, including B naive, B memory and PCs, had increased infiltration in LUAD (Figure 5E). We performed an analysis of XBP1 expression in B lymphocytes and found that XBP1 was not only highly expressed in PCs (Figures 5F,G), but also showed higher expression in infiltrating PCs compared to normal PCs (Figure 5H). In addition, we projected the pathway scores of two gene sets (GOBP: Negative regulation of response to endoplasmic reticulum stress and GOBP: protein folding) onto the single-cell data and found that both scores were increased in infiltrating PCs, suggesting enhanced functional activity of ERS and protein folding in infiltrating PCs (Figure 5I,J).

### The contribution of X-box binding protein 1 isoforms to the survival of lung adenocarcinoma patients

**FIGURE 6:**
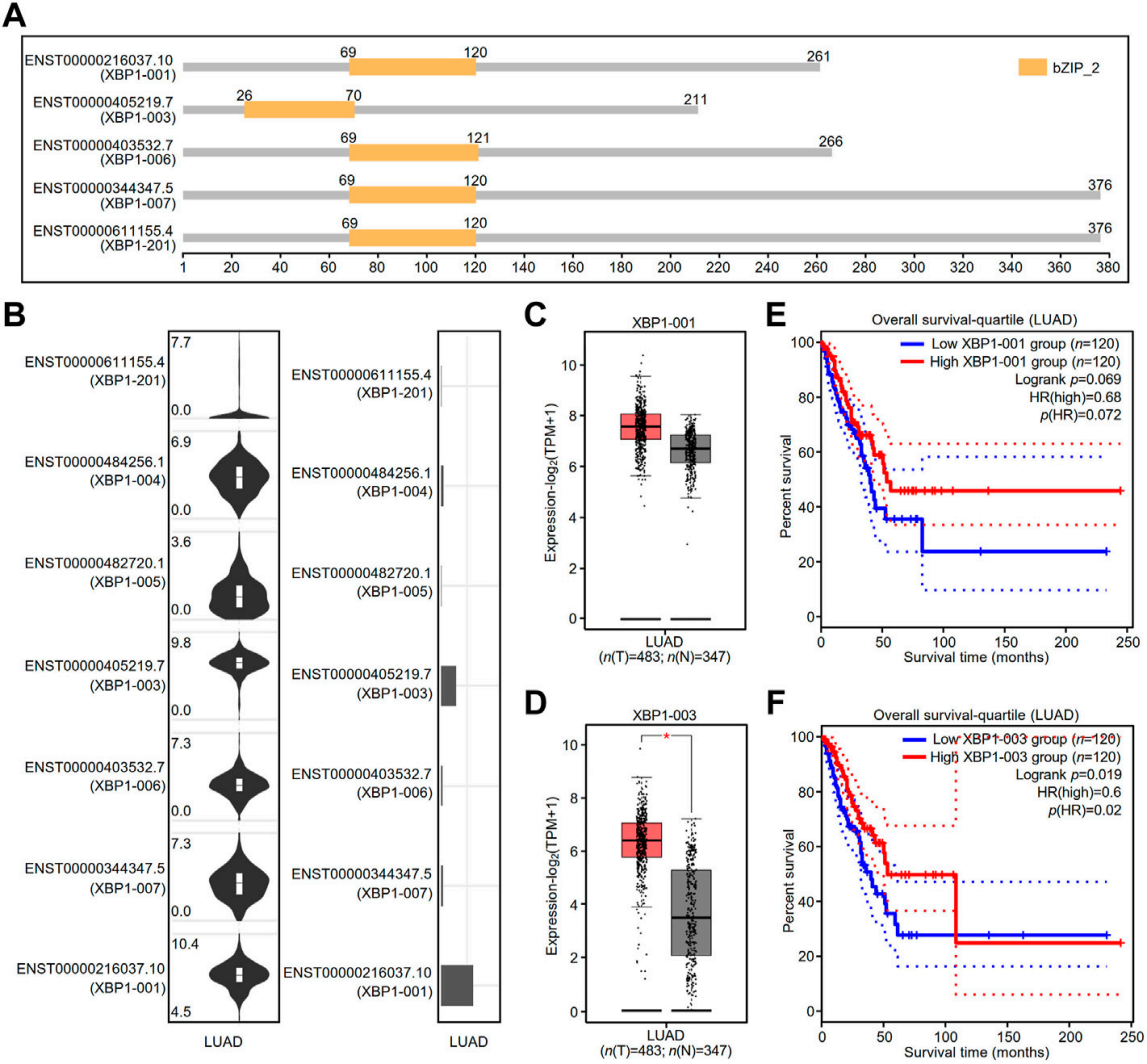
The expression and prognostic value of XBP1 isoforms in LUAD. **(A)** XBP1 isoforms and the plot for their protein domain structures. **(B)** The expression distribution (violin plot) and isoform usage (bar plot) of XBP1 in LUAD. **(C,D)** The expression of XBP1 isoforms XBP1-001 **(C)** and XBP1-003 **(D)** in LUAD. (*p < 0.05) **(E,F)** The prognostic value for overall survival of LUAD patients of XBP1 isoforms XBP1-001 **(E)** and XBP1-003 **(F)**.

The GEPIA2 database showed that there were at least five splicing isoforms of XBP1 in LUAD (Figure 6A). As shown in the violin and bar plots, two splicing isoforms, XBP1-001 and XBP1-003, were the most abundant transcripts in LUAD (Figure 6B).

We thus examined the mRNA expression of XBP1 and found that the expression of the transcript of XBP1-003, but not XBP1-001, was significantly higher in LUAD tissues than in normal tissues, suggesting that XBP1-003 was induced to be produced in LUAD (Figures 6C,D). In addition, we evaluated the prognostic value of XBP1 isoforms and found that only high XBP1-003 expression was significantly associated with a good prognosis in LUAD patients (Figures 6E,F).

### Unique function of X-box binding protein 1-003 in the progression of lung adenocarcinoma

**FIGURE 7:**
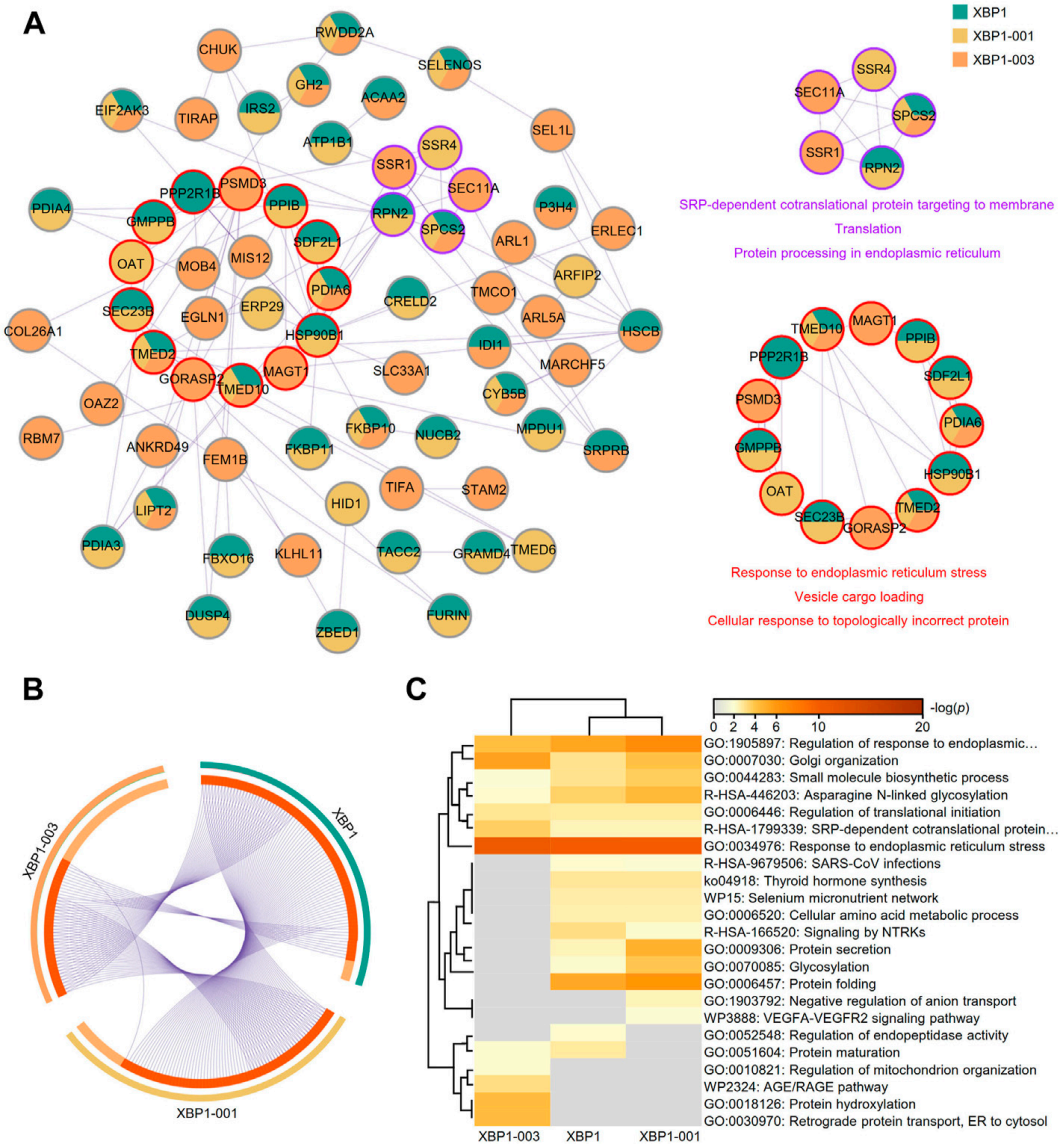
Metascape analysis of the top 100 genes with similar expression profiles of XBP1 isoforms in LUAD. **(A)** The PPI network and two modules (purple and red) with significance were obtained by Metascape analysis. **(B)** Overlap of the top 100 genes with similar expression profiles of total XBP1, XBP1-001 and XBP1-003 in LUAD. **(C)** Heatmap of the top 100 genes with similar expression profiles of total XBP1, XBP1-001 and XBP1-003 in LUAD, colored by *p*-values.

Based on the finding of a correlation between XBP1-003 and a good prognosis in LUAD patients, we used mixed genes with profiles similar to those of XBP1 isoforms in Metascape to further analyze the different functions of XBP1 isoforms in LUAD. The PPI network was composed of 68 proteins representing 105 protein-protein interaction pairs, of which 36.76% (25/68) proteins were exclusively identified as similar to XBP1-003, suggesting that XBP1-003 plays a distinct important role in LUAD (Figure 7A). Functional enrichment analysis revealed two core modules in the PPI network; the purple module was found to be involved in protein translation and protein processing, while the red module might participate in the response to ERS and vesicle cargo loading (Figure 7A).

A Venn diagram showed that XBP1-001 had more similar genes overlapping with XBP1 than XBP1-003, which indicates that XBP1-003 is unique and performs a distinct function in improving the survival of LUAD patients (Figure 7B). Different XBP1 isoforms can share some biological functions; for example, the XBP1-001 and XBP1-003 isoforms were both involved in the regulation of ERS in LUAD. Interestingly, we found that XBP1-003 specifically participated in the regulation of the AGE/RAGE pathway as well as BPs, including protein hydroxylation, regulation of mitochondrial organization and ER to cytosol retrograde protein transport (Figure 7C).

### Structural basis for X-box binding protein 1-003 antitumorigenic function

**FIGURE 8:**
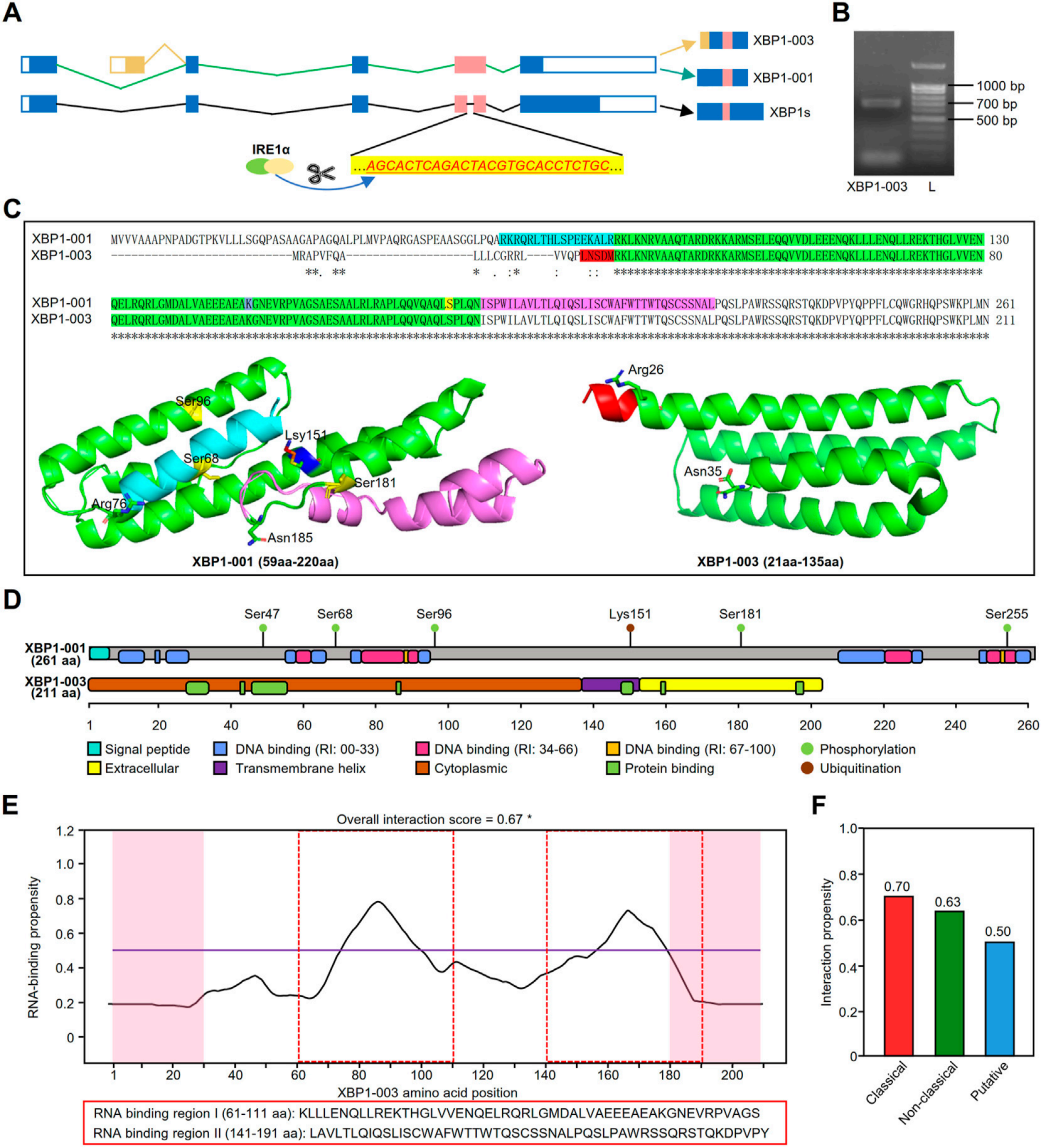
Structural and functional prediction of XBP1 isoforms. **(A)** The schematic of XBP1 pre-mRNA where the small intron (26 nt) recognized and excised by IRE1α in red. **(B)** Gel electrophoresis showed that the amplification products from MM.1S. L, ladder. **(C)** Pairwise alignment of amino acid sequences of XBP1-001 (ID: P17861) and XBP1-003 (ID: B1AHH1) in UniProt database and three dimensional structure of XBP1-001 (59aa-220aa) and XBP1-003 (21aa-135aa) protein fragments. The protein data with PDB IDs 5a7dL and 2yfaA were downloaded from the ModBase. **(D)** The schematic diagram of protein domains and post-translational modification sites of XBP1-001 and XBP1-003. Specific domains are shown in distinct colors. The protein domains were predicted with PredictProtein, and information of phosphorylation/ubiquitination was collected from the cBioPortal. **(E)** Prediction of RNA binding-sites of XBP1-001 protein by catRAPID. *: prediction score >0.5. **(F)** Interaction propensity scores for the RNA binding-sites of XBP1-003 derived from three independent methods in catRAPID.

The schematic of XBP1 pre-mRNA showed that the XBP1-001 and XBP1-003 isoforms have variation in the N-terminal coding sequences as a result of alternative splicing (Figure 8A). To prove the real existence of XBP1-003, we amplified the PCR product and found that XBP1-003 (671 bp) was indeed present in PCs (Figure 8B). Multiple sequence alignment of sequencing results further confirmed the presence of XBP-003 in PCs (Supplementary Figure S5A).

A pairwise comparison of protein sequences in UniProt revealed that the XBP1-001 and XBP1-003 sequences were different in the N-terminus, but the remaining major sequences were identical (Figure 8C). Three-dimensional structure data downloaded from ModBase in PyMOL showed that although the primary sequence of the green portion of XBP1 was identical, the structures of these two isoforms were significantly different. XBP1-001 contains four α-helices, i.e., α1 (81–105 aa), α2 (114–123 aa), α3 (127–163 aa) and α4 (167–181 aa), whereas XBP1-003 contains only three α-helices, α1 (26–54 aa), α2 (59–88 aa) and α3 (92–135 aa) (Figure 8C).

To further clarify whether the special structure affects the functional domains, we used PredictProtein to predict the protein domains of these XBP1 isoforms. Intriguingly, we found that the XBP1-001 protein contains several DNA binding sites and a signal peptide (SP) at the N-terminus, while the XBP1-003 protein indeed only has a transmembrane structure and a protein binding site (Figure 8D).

GO analysis in PredictProtein showed that both XBP1-001 and XBP1-003 were associated with some BP terms, such as protein processes and cell differentiation. Consistent with the results above, we found that only XBP1-003 was able to positively regulate PC differentiation and immunoglobulin production (Supplementary Tables S1,S2). Additional evidence of posttranslational modification supports the hypothesis that these XBP1 isoforms have different functions in LUAD. Information from cBioPortal showed that XBP1-001 has five phosphorylation sites and one ubiquitination site, but XBP1-003 does not (Figure 8D).

To further differentiate the biological functions of XBP1-003 from XBP1-001, we performed RNA binding site prediction on the catRAPID website and found that XBP1-003, but not XBP1-001, had two RNA binding sites with an overall interaction score higher than 0.5 (Figure 8E). Three independent and class-specific scores of the putative RNA binding sites provide more detailed information to support the predicted results above (Figure 8F).

## Discussion

XBP1 is located on chromosome 22q12.1 (Supplementary Figure S6A) and is known for its role in ERS induced by the accumulation of unfolded or misfolded proteins. It is well established that XBP1 is involved in ERS to maintain intracellular homeostasis and promote cell survival (Xu et al., 2021), but the detailed molecular mechanisms remain to be illustrated.

Notably, many studies have demonstrated that XBP1, which has a close association with tumorigenesis and progression, is involved in the intense competition for nutrients and metabolites between cancer cells and TILs in the TME (Buck et al., 2017). We found that a high abundance of infiltrated B cells was positively correlated with XBP1 overexpression and associated with a better prognosis in LUAD patients, which is consistent with previous studies showing that the quantity of TIBs is a key factor in tumor immunity and is associated with longer survival in LUAD patients (Wei et al., 2019). Moreover, XBP1 has been reported to participate in the immune system pathway in LUAD (Liu et al., 2018). Here, we propose that XBP1 expression promotes B cell differentiation and antibody secretion from PCs in LUAD, thus bringing about a substantial change in the TME that is favorable in terms of the prognosis of LUAD. Indeed, there is evidence that the XBP1 signaling pathway contributes to PC differentiation and survival mediated by protein homeostasis (Aragon et al., 2012). Shaffer et al. (2004) also found that XBP1 plays a profound role in the antibody secretory capacity of PCs by promoting the expression of genes involved in ER homeostasis and by driving an increase in cell size. It has also been shown that antibody secretion is severely reduced in XBP1^−/−^ PCs (Tellier et al., 2016). This indicates that XBP1 performs a biological function in B cells. Furthermore, Isaeva et al. (2019) reported that the presence of intratumoral IgG1 is typically associated with a good prognosis in many cancer types as it drives complement activation, phagocytosis and antibody-dependent cell cytotoxicity (ADCC), but a high level of IgG4 in tumors is commonly linked with a poor prognosis as it interferes with the positive effects of tumor-specific IgG1. It has also been reported that cholecystokinin octapeptide (CCK-8) inhibits IgG1 production of B cells by a mechanism that may be associated with XBP1 (Zhang et al., 2011). Shortly after that, Benhamron et al. (2014) also demonstrated that lgG1 production was inhibited in XBP1 knockout mice. Therefore, we suggest that high expression of XBP1 alleviates ERS caused by TME in B cells and promotes antitumorigenic antibody production in PCs, thus resulting in the improvement of overall survival in LUAD patients.

Studies have suggested that B cells may form structured TLSs by forming tumor-associated immune aggregates of various complexity and that this structure has prognostic value in varying metastatic and primary tumors (Sautès-Fridman et al., 2019). In particular, antigen-specific interactions between T cells and B cells in the TLS seem to be crucial, and the protective role of T cells in the TME often depends on cooperation with B cells (Zhong et al., 2021). It has been found that B cells can maintain additional T cell population expansion intratumorally by acting as APCs compared to dendritic cells (DCs) that provide initial T cell activation (Bruno et al., 2017). This suggests that B cell infiltration in LUAD patients performs antitumor functions, perhaps relying on cooperation with T cell populations. In addition, monalizumab as an immunotherapeutic agent that stimulates NK cells contributes to a more pronounced response for patients with highly produced IgG1 of tumor-associated PCs (André et al., 2018). Notably, several studies activating NK cell populations with anti-PD1 therapy found that the combination of B cell-promoting and PD1-blocking therapies could provide antitumor effects owing to synergistic effects on B and NK cells (Hsu et al., 2018; Sanchez-Correa et al., 2019). This implies that the B cell infiltration in LUAD may be associated with the NK cell population as well. Moreover, new research shows that SETDB1 fuels the phenotype of lung cancer by modulating the epigenome and resists anti-CTLA4 and anti-PD1 combination therapy (Griffin et al., 2021; Zakharova et al., 2022). Pasquarella et al. (2016) also detected an increased level of Xbp1 expression in SETDB1-deficient B cells. Therefore, we have a reason to speculate that XBP1 expression may be linked to CTLA4 and PD1.

Previous studies have demonstrated that there are several alternative splicing isoforms of XBP1 in humans. For example, the long splicing isoform of XBP1 (XBP1s) is produced by excision of a small intron (26 nt) from the XBP1 pre-mRNA transcript, which is mediated by RNase activation induced by IRE1α autophosphorylation in ERS (Figure 8A), whereas the transcript for XBP1u, caused by retention of the excised 26 nt sequence, encodes a short isoform due to the early stoppage of mRNA translation (Uemura et al., 2013). Zhang et al. (2021) found that XBP1u mRNA expression was higher than XBP1s mRNA expression in LUAD, indicating that XBP1u mRNA has a stronger effect on LUAD cells. We compared the TaqMan probe sequences of XBP1s and XBP1u obtained from several independent studies with all sequences of XBP1 isoforms in the NCBI database and unexpectedly found that the probes of XBP1u were unable to distinguish the two transcripts of XBP1, i.e., XBP1-001 and XBP1-003 (Supplementary Figure S6B) (Davies et al., 2008; Fink et al., 2018). After careful comparison of the protein sequences of XBP1-001 and XBP1-003, we found that XBP1-003 represents a novel splicing variant. And we also confirmed the existence of XBP1-003 experimentally for the first time.

To investigate the role of XBP1 isoforms in LUAD, we assessed the gene expression of XBP1 isoforms and found that XBP1-001 had the highest expression. However, XBP1-001 expression was not different between LUAD tissues and adjacent normal tissues, and there was also no difference in the survival of patients with high versus low expression of XBP1-001. Intriguingly, we found that XBP1-003 expression is significantly increased in LUAD samples compared to normal samples and is closely associated with a good prognosis in LUAD patients. These results suggest that alternative splicing of XBP1 mRNA improves the prognosis of LUAD patients.

Given the significance of XBP1-003 in LUAD, it is important to clarify the molecular mechanisms involved in XBP1 gene splicing. A previous study showed that the UPR promotes the homo-oligomerization of IRE1α, induces its trans-autophosphorylation, and further drives the activation of kinase/endonuclease (RNase) to mediate XBP1 mRNA splicing (Ghosh et al., 2014). Wang et al. (2015) reported that IRE1α is involved in unconventional mRNA splicing by removing the 26 nt intron within the XBP1 mRNA open reading frame to generate XBP1s Protein sequence alignment showed that the XBP1-003 transcript has alternative splicing on the first exon at the N-terminal coding sequence but without 26 nt exon splicing, similar to XBP1-001 (Figure 8C). Therefore, it remains to be investigated whether the splicing of XBP1-003 is also mediated by IRE1α.

Despite the fact that the sequences of XBP1-001 and XBP1-003 are identical in the conserved parts, the N-terminal amino acid sequences of these two isoforms are completely different with the alternative usage of the first exon (Figure 8A). Structural information analysis demonstrated that the alternative splicing of XBP1-003 leads to a three-dimensional structure completely different from that of XBP1-001, leaving only XBP1-003 with protein binding sites, RNA binding sites and transmembrane structures, which might be the basis for the unique function of XBP1-003.

Furthermore, F-box/WD repeat-containing protein 7 (FBW7) was found to promote XBP1 ubiquitination and protein degradation in a phosphorylation-dependent manner (Chae et al., 2019); PTEN-induced kinase 1 (PINK1) was reported to induce phosphorylation of XBP1s (El Manaa et al., 2021). These results indicate that phosphorylation and ubiquitination are involved in the regulation of XBP1 function. Our bioinformatics analysis showed that XBP1-001 has two highly potential phosphorylation sites (Ser^47^ and Ser^68^) at the N-terminus of the peptide chain, while XBP1-003 does not contain this sequence, thus providing supporting evidence for the specific function of this newly identified isoform of XBP1. We also found that although both XBP1 isoforms have a lysine site in the conserved region, only XBP1-001 shows potential regulation by ubiquitination on Lys^151^, indicating that XBP1-003 has a special spatial structure due to the lack of an N-terminal fragment. However, further studies are needed to illuminate the detailed mechanism to explain the differences between them.

Both inflammation and tumors have been reported to induce hostile environments, driving the UPR of the ER (Zschaeck et al., 2021). Indeed, the UPR is thought to be involved in the regulation of mitochondrial function in tumorigenesis and progression (Bhat et al., 2017). It is well established that RAGE is involved in promoting mitochondrial adenosine triphosphate (ATP) production and inducing mitochondrial autophagy (Daussin et al., 2021). Interestingly, in our research, further evaluation of the potential role of XBP1 isoforms showed that XBP1-003 participates in the regulation of the AGE/RAGE pathway, mitochondrial organization, and ERS in LUAD (Figure 7C). A study on a diabetic nephropathy (DN) model demonstrated that activation of AGE/RAGE signaling stimulates NADPH oxidase-mediated production of ROS, which subsequently activates the UPR, resulting in the alleviation of ERS (Pathomthongtaweechai and Chutipongtanate, 2020). Moreover, it has also been shown that RAGE is highly expressed in B lymphocytes and is associated with B cell activation (Sparvero et al., 2009). B cells in undernourished and hypoxic TME initiate ERS. Thus, it is reasonable to assume that alternative splicing of XBP1 in B cells may initiate ERS supported by AGE/RAGE pathways and mitochondrial ATP, thereby promoting B cell resistance to stress.

For the first time, we found that the novel isoform XBP1-003 is not only a distinctive factor associated with the survival of patients but also a key regulator of B cell infiltration in LUAD; however, this study still has some limitations. Specifically, the results presented here were from an analysis of bioinformatics data in combination with previous experimental studies aimed at reaching a reasonable conclusion on the function of XBP1-003 in PCs, but these important findings need further experimental validation. However, there might be some experimental challenges in extracting B cells from the tumor tissues of LUAD patients and in distinguishing the isoforms of XBP1 for identification of its specific function.

In summary, we applied integrated bioinformatics approaches to determine the prognostic value of different splicing isoforms of XBP1 in LUAD patients and found that XBP1 plays an antitumorigenic role in LUAD through alternative splicing, which may be linked to the adaptation of plasma cells. Our study sheds new light on the potential strategy for LUAD prognosis evaluation and immunotherapy.

## Data Availability

The original contributions presented in the study are included in the article/Supplementary Material, further inquiries can be directed to the corresponding authors.
