# Supplementary material for: XBP1 impacts lung adenocarcinoma progression by promoting plasma cell adaptation to the tumor microenvironment
Source: Front Genet. 2022 Aug 24;13:969536. doi: 10.3389/fgene.2022.969536 (PMC9448868; doi:10.3389/fgene.2022.969536)
Supplement: Supplementary file 1 [file DataSheet2.PDF]

Supplementary table 1. The biological process ontology analysis of XBP1 isoform (261aa) by PredictProtein

#	GO ID	Biological Process Term
1	GO:0030154	cell differentiation
2	GO:1903489	positive regulation of lactation
3	GO:0006633	fatty acid biosynthetic process
4	GO:0031017	exocrine pancreas development
5	GO:0045600	positive regulation of fat cell differentiation
6	GO:0042632	cholesterol homeostasis
7	GO:0006915	apoptotic process
8	GO:0070059	intrinsic apoptotic signaling pathway in response to endoplasmic reticulum stress
9	GO:0007275	multicellular organism development
10	GO:0042149	cellular response to glucose starvation
11	GO:0071353	cellular response to interleukin-4
12	GO:0051023	regulation of immunoglobulin production
13	GO:0001889	liver development
14	GO:0036500	ATF6-mediated unfolded protein response
15	GO:0006990	positive regulation of transcription from RNA polymerase II promoter involved in unfolded protein response
16	GO:0050714	positive regulation of protein secretion
17	GO:0050707	regulation of cytokine production
18	GO:0071222	cellular response to lipopolysaccharide
19	GO:0006366	transcription by RNA polymerase II
20	GO:0048666	neuron development

Supplementary table 2. The biological process ontology analysis of XBP1 isoform (211aa) by PredictProtein

#	GO ID	Biological Process Term
1	GO:0006511	ubiquitin-dependent protein catabolic process
2	GO:0015031	protein transport
3	GO:0060691	epithelial cell maturation involved in salivary gland development
4	GO:0007517	muscle organ development
5	GO:1900100	positive regulation of plasma cell differentiation
6	GO:0031670	cellular response to nutrient
7	GO:0032755	positive regulation of interleukin-6 production
8	GO:1990418	response to insulin-like growth factor stimulus
9	GO:0051024	positive regulation of immunoglobulin production
10	GO:0030154	cell differentiation
11	GO:1900103	positive regulation of endoplasmic reticulum unfolded protein response
12	GO:0050715	positive regulation of cytokine production
13	GO:0010832	negative regulation of myotube differentiation
14	GO:0006914	autophagy
15	GO:0001935	endothelial cell proliferation
16	GO:0060612	adipose tissue development
17	GO:0010506	regulation of autophagy
18	GO:0071332	cellular response to fructose stimulus
19	GO:0055081	anion homeostasis
20	GO:2000347	positive regulation of hepatocyte proliferation
