# Supplementary material for: XBP1 impacts lung adenocarcinoma progression by promoting plasma cell adaptation to the tumor microenvironment
Source: Front Genet. 2022 Aug 24;13:969536. doi: 10.3389/fgene.2022.969536 (PMC9448868; doi:10.3389/fgene.2022.969536)
Supplement: Supplementary file 2 [file DataSheet1.PDF]

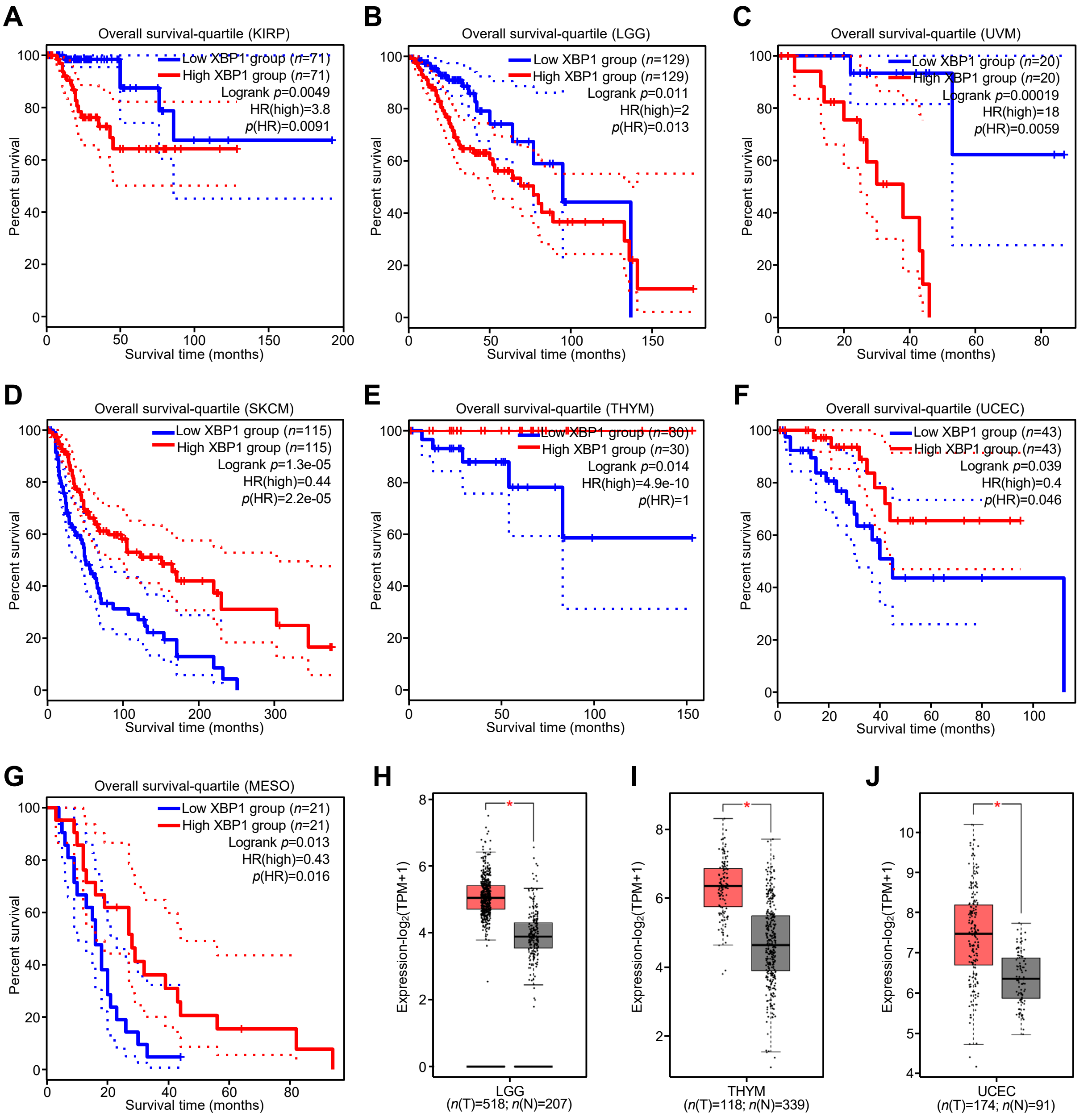

Supplementary Figure 1. Kaplan-Meier survival curves for high (red) and low (blue) expression of XBP1 in different tumor types and boxplots showing relative expression in different tumors and normal controls in the GEPIA2.

(A-G) Survival curves of OS with significance in seven tumor types (KIRP, LGG, UVM, SKCM, THYM, UCEC, and MESO). **(I-J)** Expression of XBP1 with significance in three of the seven tumors (LGG, THYM, and UCEC). (*p<0.05)

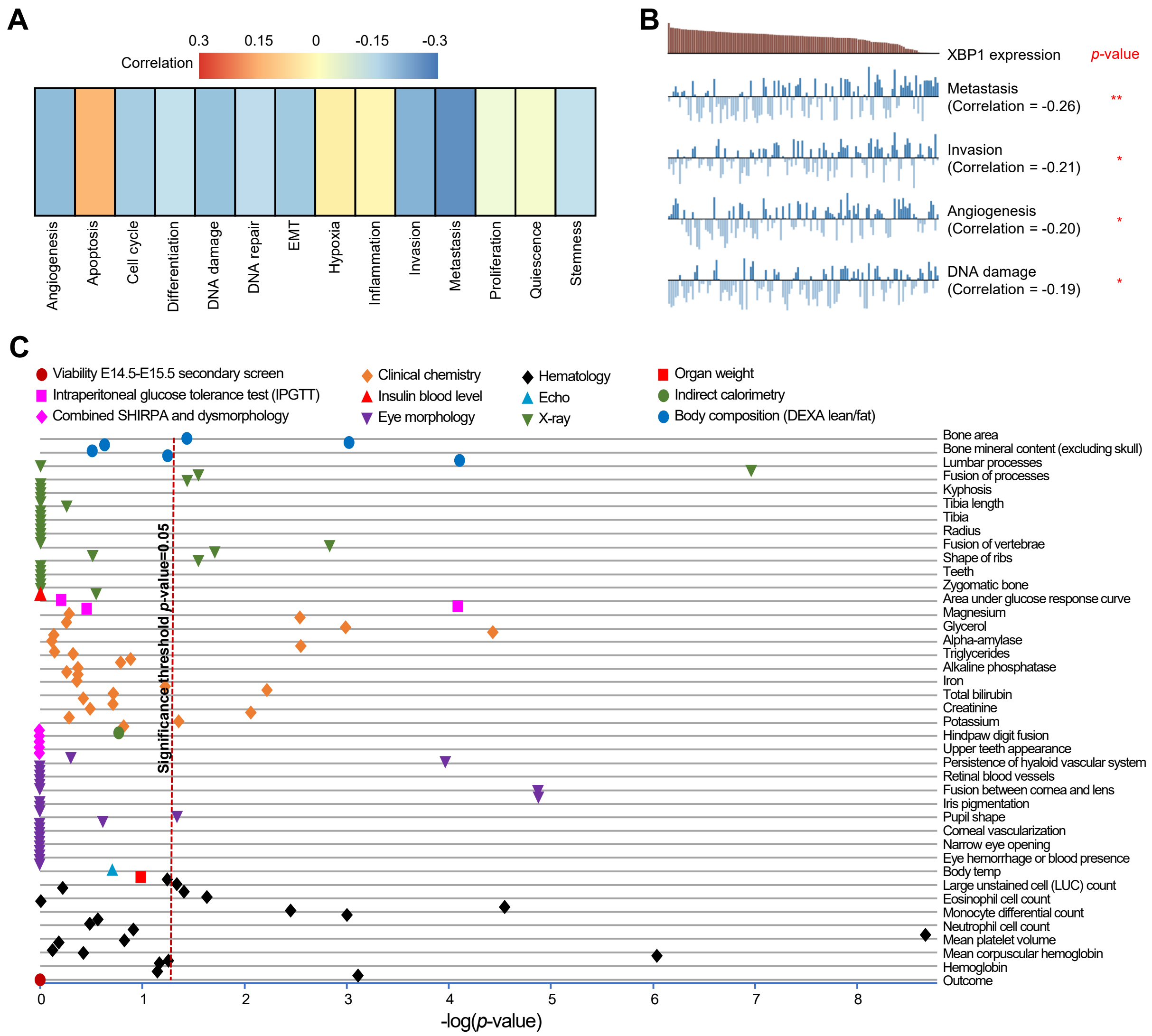

Supplementary Figure 2. Functional analysis of XBP1.

(A) Functional relevance analysis of XBP1 in LUAD using cancerSEA. Color represents the average correlation strength. **(B)** Detailed functional correlations in LUAD. **(C)** Parameters of significant phenotype changes in mice associated with XBP1 were measured by International Mouse Phenotyping Consortium (IMPC). (*$p<0.05$ and **$p<0.01$)

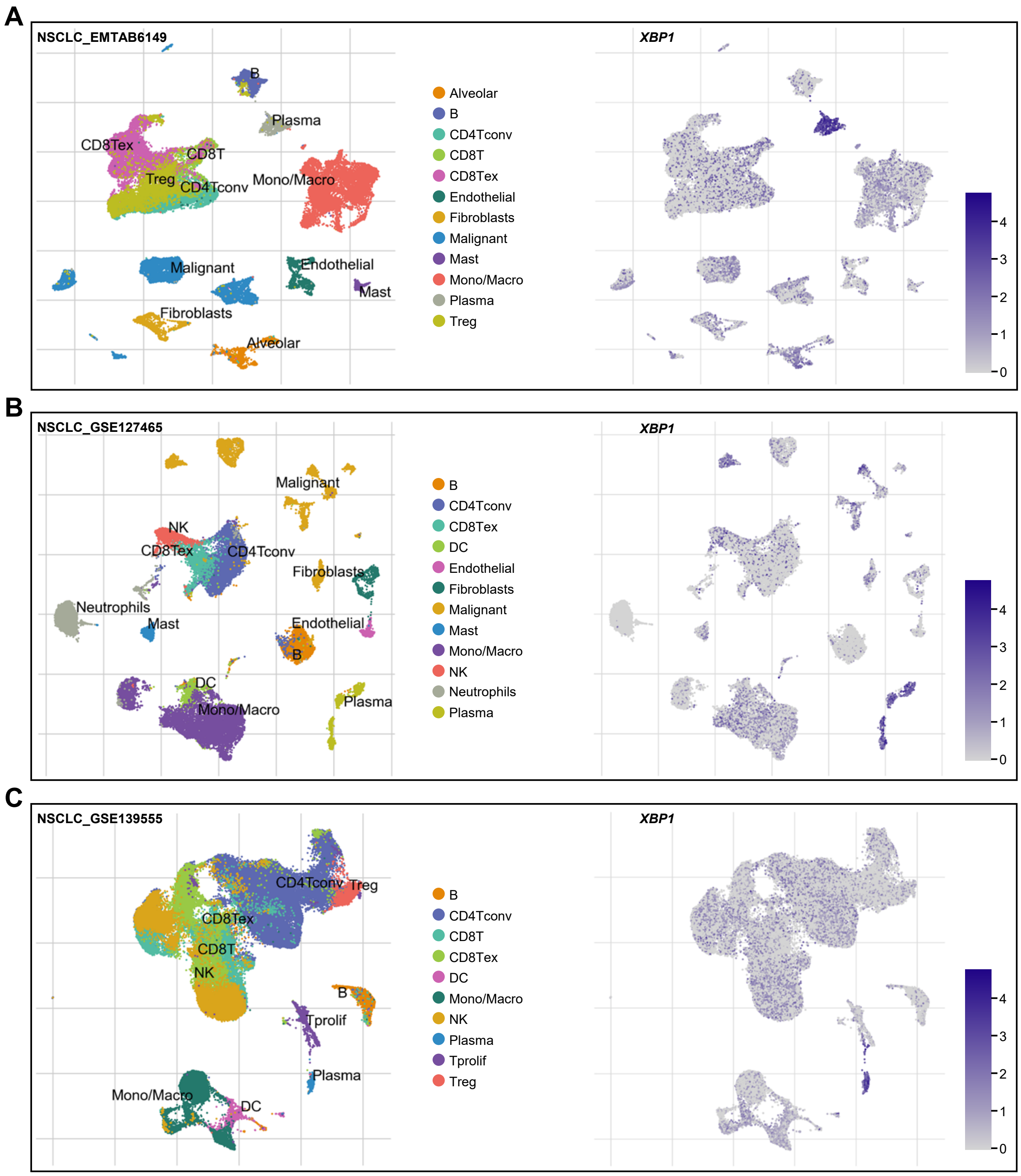

Supplementary Figure 3. Single-cell RNA sequencing analysis.

Expression levels of XBP1 were analyzed by Tumor Immune Single-cell Hub (TISCH) in 3 different datasets, including NSCLC_EMTA6149 **(A),** NSCLC_GSE127465 **(B)** and NSCLC_GSE139555 **(C).** Expression levels were colored by marker intensity.

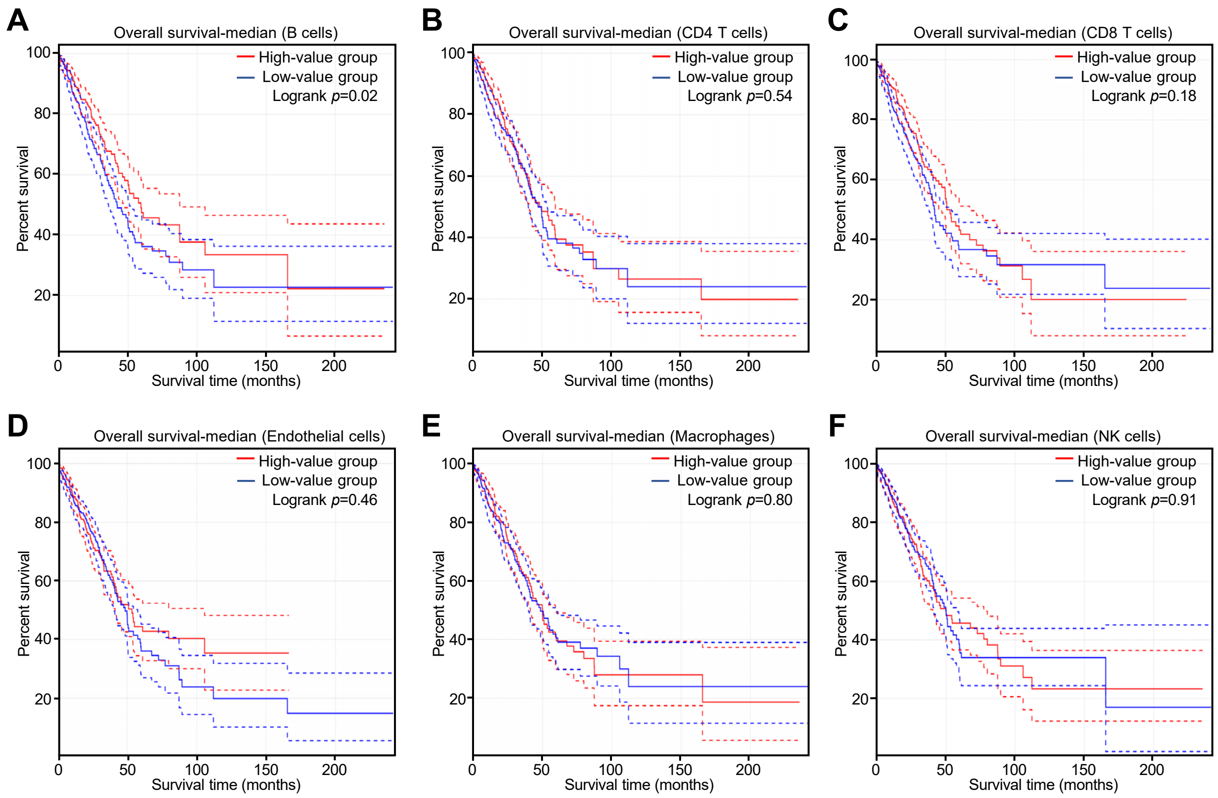

Supplementary Figure 4. Kaplan-Meier survival curves for tumor-infiltration immune cells in LUAD.

Overall survival curves for high (red) and low (blue) infiltration of B cells **(A)**, CD4 T cells **(B)**, CD8 T cells **(C)**, endothelials **(D)**, macrophages **(E)** and NK cells **(F)** in LUAD were plotted by GEPIA2021 according to the EPIC. The statistical difference between the curves was measured by the log-rank test.

A

```
multiple sequence alignment

XBP1-003           CCGGACTGACCGGATCCGCCACGCTGGGAACCTAGGGCGGCCCAGGGCTCTTTTCTGTACTTTTTAACTCTCTCGTTAGAGATGACCAGAGCTGGGGATGCGGGCACCTGTCTTCCAGGC  120
sequencing results -------------------------------------------------------------------------CGTTAGAGATGACCAGAGCTGGGGATGCGGGCACCTGTCTTCCAGGC  47
                                                                                            ***********************************************

XBP1-003           CCTCTTGCTGTGTGGCCGCAGACTGGTGGTTCAGCCTCTTAACTCGGACATGAGGAAACTGAAAAACAGAGTAGCAGCTCAGACTGCCAGAGATCGAAAGAAGGCTCGAATGAGTGAGCT  240
sequencing results CCTCTTGCTGTGTGGCCGCAGACTGGTGGTTCAGCCTCTTAACTCGGACATGAGGAAACTGAAAAACAGAGTAGCAGCTCAGACTGCCAGAGATCGAAAGAAGGCTCGAATGAGTGAGCT  167
                   ************************************************************************************************************************

XBP1-003           GGAACAGCAAGTGGTAGATTTAGAAGAAGAGAACCAAAAACTTTTGCTAGAAAATCAGCTTTTACGAGAGAAAACTCATGGCCTTGTAGTTGAGAACCAGGAGTTAAGACAGCGCTTGGG  360
sequencing results GGAACAGCAAGTGGTAGATTTAGAAGAAGAGAACCAAAAACTTTTGCTAGAAAATCAGCTTTTACGAGAGAAAACTCATGGCCTTGTAGTTGAGAACCAGGAGTTAAGACAGCGCTTGGG  287
                   ************************************************************************************************************************

XBP1-003           GATGGATGCCCTGGTTGCTGAAGAGGAGGCGGAAGCCAAGGGGAATGAAGTGAGGCCAGTGGCCGGGTCTGCTGAGTCCGCAGCACTCAGACTACGTGCACCTCTGCAGCAGGTGCAGGC  480
sequencing results GATGGATGCCCTGGTTGCTGAAGAGGAGGCGGAAGCCAAGGGGAATGAAGTGAGGCCAGTGGCCGGGTCTGCTGAGTCCGCAGCACTCAGACTACGTGCACCTCTGCAGCAGGTGCAGGC  407
                   ************************************************************************************************************************

XBP1-003           CCAGTTGTCACCCCTCCAGAACATCTCCCCATGGATTCTGGCGGTATTGACTCTTCAGATTCAGAGTCTGATATCCTGTTGGGCATTCTGGACAACTTGGACCCAGTCATGTTCTTCAAA  600
sequencing results CCAGTTGTCACCCCTCCAGAACATCTCCCCATGGATTCTGGCGGTATTGACTCTTCAGATTCAGAGTCTGATATCCTGTTGGGCATTCTGGACAACTTGGACCCAGTCATGTTCTTCAAA  527
                   ************************************************************************************************************************

XBP1-003           TGCCCTTCCCCAGAGCCTGCCAGCCTGGAGGAGCTCCCAGAGGTCTACCCAGAAGGACCCAGTTCCTTACCAGCCTCCCTTTCTCTGTCAGTGGGGACGTCATCAGCCAAGCTGGAAGCC  720
sequencing results TGCCCTTCCCCAGAGCCTGCCAGCCTGGAGGAGCTCCCAGAGGTCTACCCAGAAGGACCCAGTTCCTTACCAGCCTCCCTTTCTCTGTCAGTGGGGACGTCATCAGCCAAGCTGGAAGCC  647
                   ************************************************************************************************************************

XBP1-003           ATTAATGAACTAATTCGTTTTGA 743
sequencing results ATTAATGAA-------------- 656
                   *********
```

Supplementary Figure 5. Sequencing and multiple-sequence alignment.

(A) The sequencing results are consistent with the XBP1-003 sequences in the database.

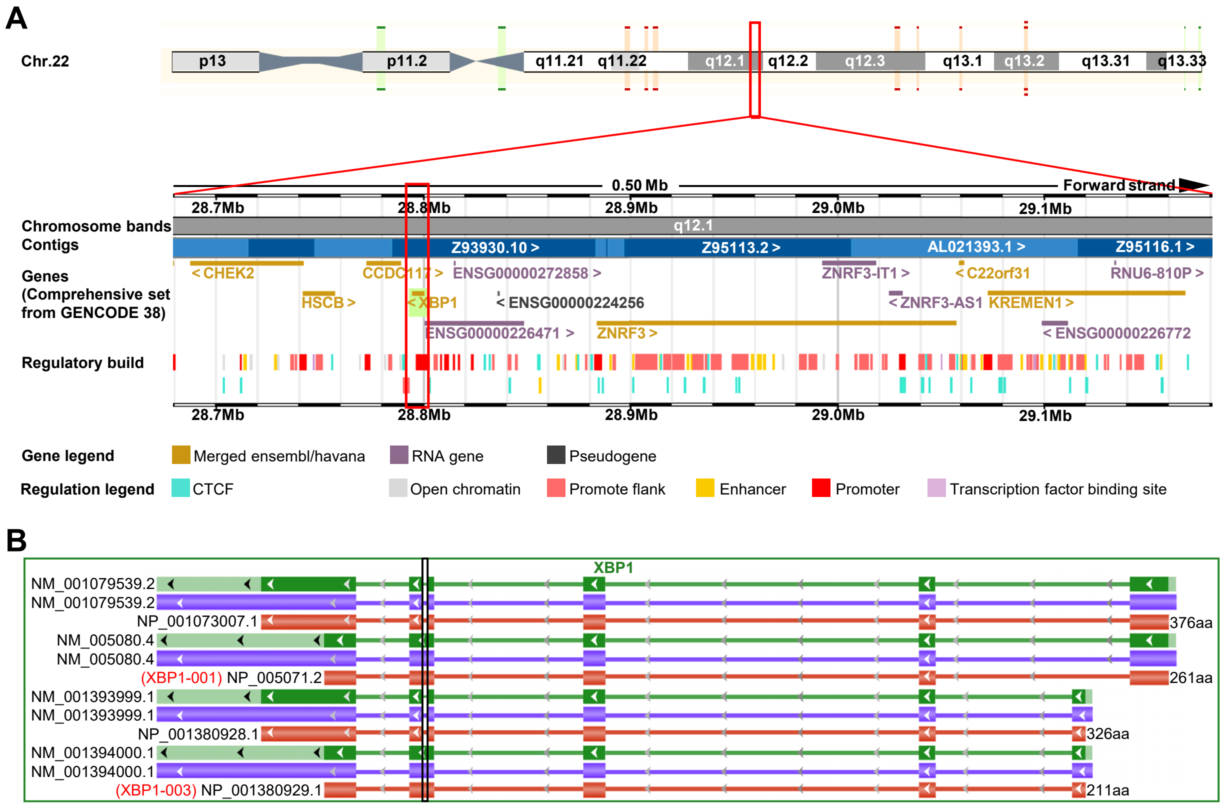

Supplementary Figure 6. The location of XBP1 and splicing-specific Taqman probes location.

(A) Location of XBP1 in the Ensembl database. **(B)** A splicing-specific Taqman probe in the black box was used to differentiate XBP1 isoforms.
